# Supplementary material for: PACT prevents aberrant activation of PKR by endogenous dsRNA without sequestration
Source: Nat Commun. 2025 Apr 8;16:3325. doi: 10.1038/s41467-025-58433-x (PMC11978871; doi:10.1038/s41467-025-58433-x)
Supplement: Supplementary file 1 — Supplementary Information [file 41467_2025_58433_MOESM1_ESM.pdf]

Supplementary Figures:

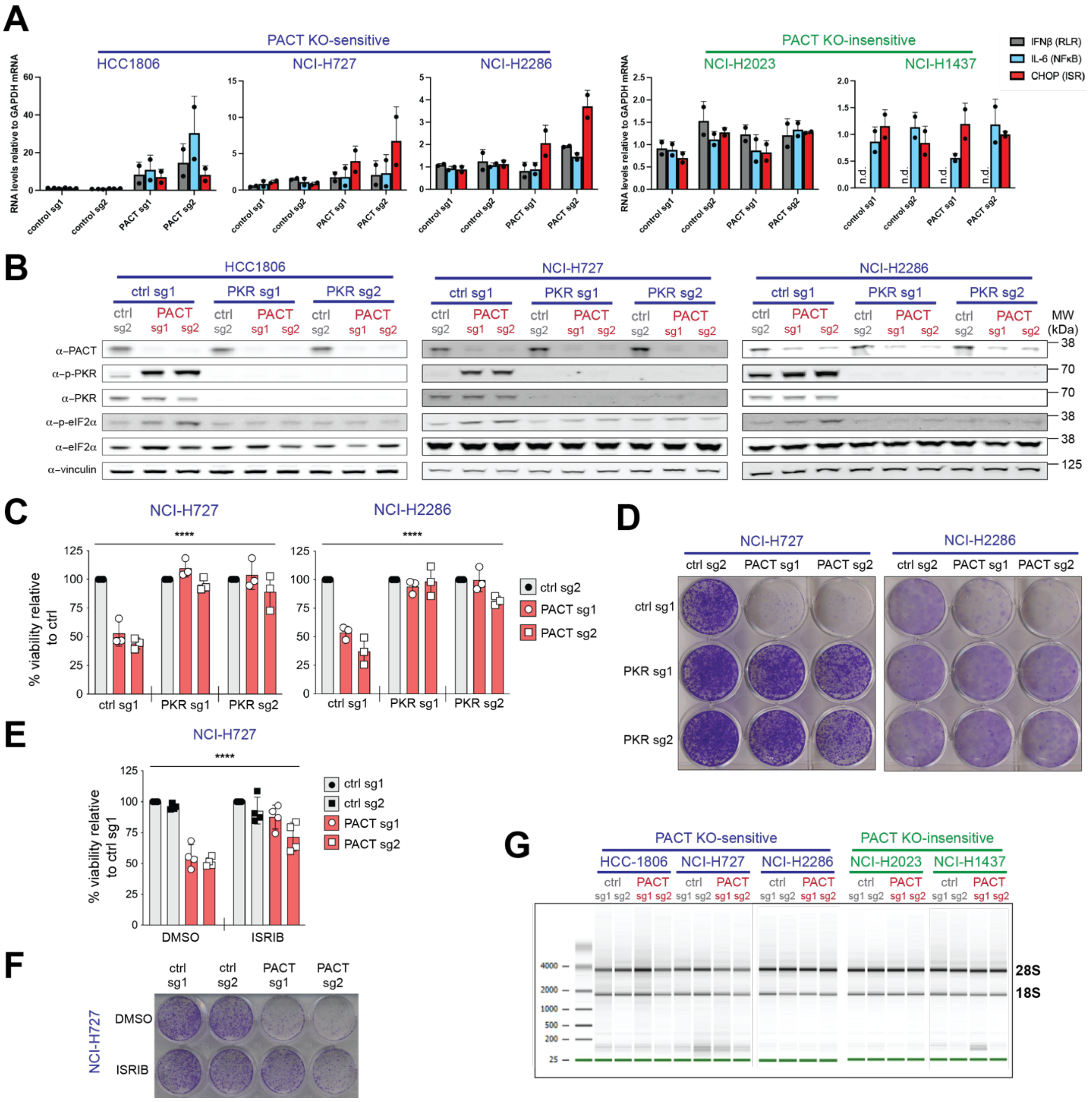

Supplementary Figure 1. PACT restricts aberrant activation of PKR, thereby maintaining cellular homeostasis.

(A) RT-qPCR analysis with RNAs extracted from PACT KO-sensitive (HCC1806, NCI-H727, NCI-H2286) and PACT KO-insensitive (NCI-H2023 and NCI-H1437) cells after control gene-KO and PACT-KO using 2 different guide RNAs. *IFNβ*, *IL-6* and *CHOP* mRNA levels relative to *GAPDH* were measured. The control genes are AAVS1 (sg1) and Chr2.2 (sg2). Values are mean (± SD) of 2 biological repeats

(B) Western blot analysis showing levels of PKR and eIF2α phosphorylation in HCC1806, NCI-H727, NCI-H2286 cells (PACT KO-sensitive cells) after knocking out indicated genes. Vinculin was used as a loading control.
(C) ATP bioluminescence cell viability assay with NCI-H727 and NCI-H2286 cells after knocking out indicated genes. Values represent means (± SD) of 3 biological repeats. *P*-values were based on two-way ANOVA test.
(D) A representative crystal violet staining assay result for samples in (C).
(E) ATP bioluminescence cell viability assay with NCI-H727 cells in control gene-KO vs. PACT-KO treated with 1μM ISRIB or DMSO. Values represent means (± SD) of 4 technical repeats. *P*-values were based on two-way ANOVA test.
(F) A representative crystal violet staining assay result from samples in (E).
(G) Bioanalyzer run with total RNA extracted from PACT KO-sensitive (HCC1806, NCI-H727, NCI-H2286) and PACT KO-insensitive (NCI-H2023 and NCI-H1437) cells after knocking out indicated genes.

**** p= <0.0001, *** p = <0.001, ** p = <0.01, * p = <0.05, not significant (ns) is for p > 0.05

Source data and exact *P*-values are provided as a Source Data file.

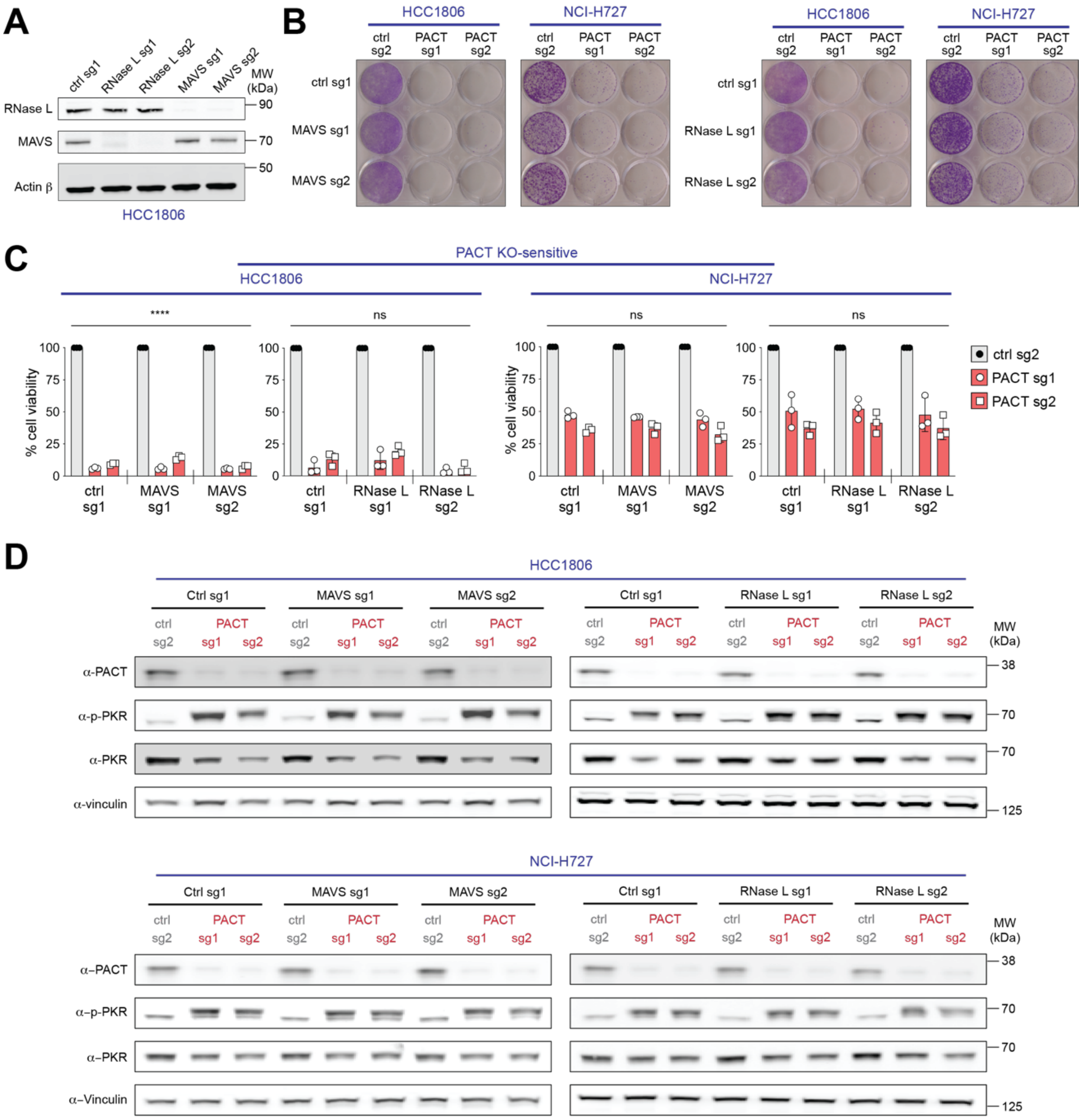

Supplementary Figure 2. PACT has little role in RLR-MAVS and OAS-RNase L pathways.

(A) Western blot analysis of HCC1806 cells after knocking out indicated genes.

(B) Crystal violet staining assay showing cell viability after knocking out indicated genes in HCC1806 and NCI-H727 cells.

(C) ATP bioluminescence assay showing cell viability after knocking out indicated genes in HCC1806 and NCI-H727 cells. Values represent means (± SD) of 3 biological repeats. *P*-values were based on two-way ANOVA test. **** *p*= <0.0001, *** *p* = <0.001, ** *p* = <0.01, * *p* = <0.05, not significant (ns) is for *p* >0.05.

(D) Western blot analysis with HCC1806 (top) and NCI-H727 (bottom) cells used in (B, C) showing PKR phosphorylation levels. Vinculin was used as loading control.

Source data and exact *P*-values are provided as a Source Data file.

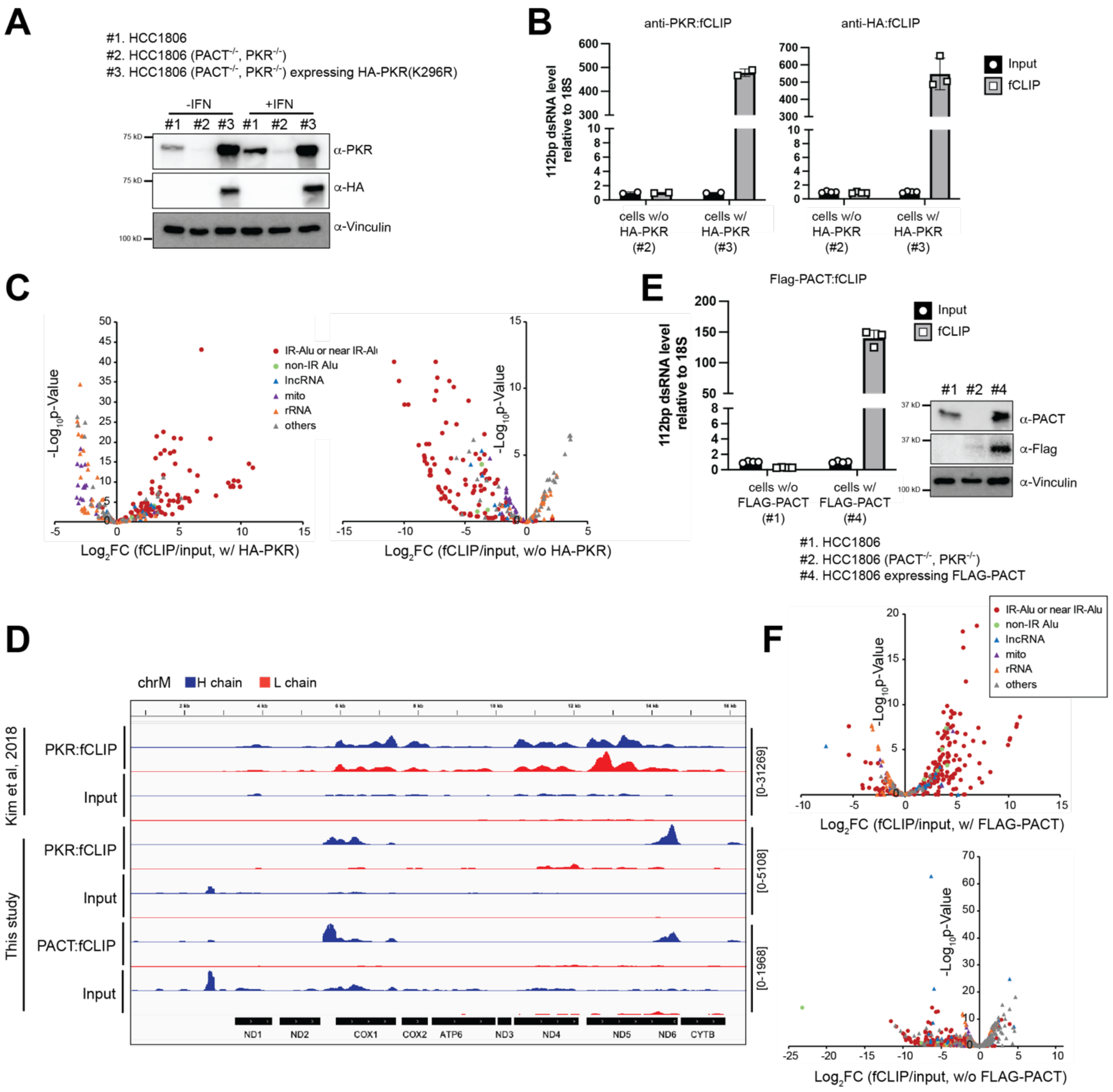

Supplementary Figure 3. PACT does not sequester endogenous dsRNA ligands away from PKR.

(A) Western blot of PKR and PACT after double knockout and complementation with catalytically dead PKR K296R in HCC1806 cells ± IFNβ (18 hr).

(B) fCLIP-RT-qPCR showing enrichment of transfected 112bp dsRNA relative to 18S rRNA in HCC1806 cells with HA-PKR (#3 from A) or without HA-PKR (#2 from A). fCLIP was performed using α-PKR or α-HA antibodies. Means (± SD) of 2 (α-PKR) and 4 (α-HA) technical replicates.

(C) Volcano plots showing peaks enriched in fCLIP-seq using HCC1806 cells expressing HA-PKR (left, #3 from A) or not (right, #2 from A). fCLIP was done using α-HA antibody. Fold change (FC) of fCLIP over input was calculated. Means of 3 biological replicates. *P*-values calculated in Deseq2 (two-sided) and adjusted by Benjamini-Hochberg method).

(D) IGV snapshots showing the mitochondrial RNA peaks from this study (PKR and PACT fCLIP) compared to the previously reported PKR fCLIP (Kim *et al*, 2018)[3]. All RNA-seq data are strand-specific, allowing separate mapping of the H and L chains. While Kim *et al*. reported a significant accumulation of the L chain, our analysis showed relatively low levels of the L chain compared to the H chain. This discrepancy may reflect cell type-dependent variability in mitochondrial RNA synthesis/metabolism (Hela in Kim *et al* vs. HCC1806 in this study) or the effect of cell-cycle arrest treatment, under which the previous study was performed.

(E) fCLIP-RT-qPCR showing enrichment of transfected 112bp dsRNA relative to 18S rRNA in HCC1806 cells with FLAG-PACT (#4 from E) or without FLAG-PACT (#1 from E). fCLIP was done using α-Flag antibody. Means (±SD) of 4 technical replicates. Right: WB showing PACT levels.

(F) Volcano plots showing peaks enriched in fCLIP-seq using HCC1806 cells expressing FLAG-PACT (top, #4 cell from A) or not (bottom, #1 cells from E). fCLIP was done using α-FLAG antibody. Fold change (FC) of fCLIP over input was calculated. Values represent means of 2 biological replicates. *P*-values calculated in Deseq2 (two-sided) and adjusted by Benjamini-Hochberg method.

Source data are provided as a Source Data file.

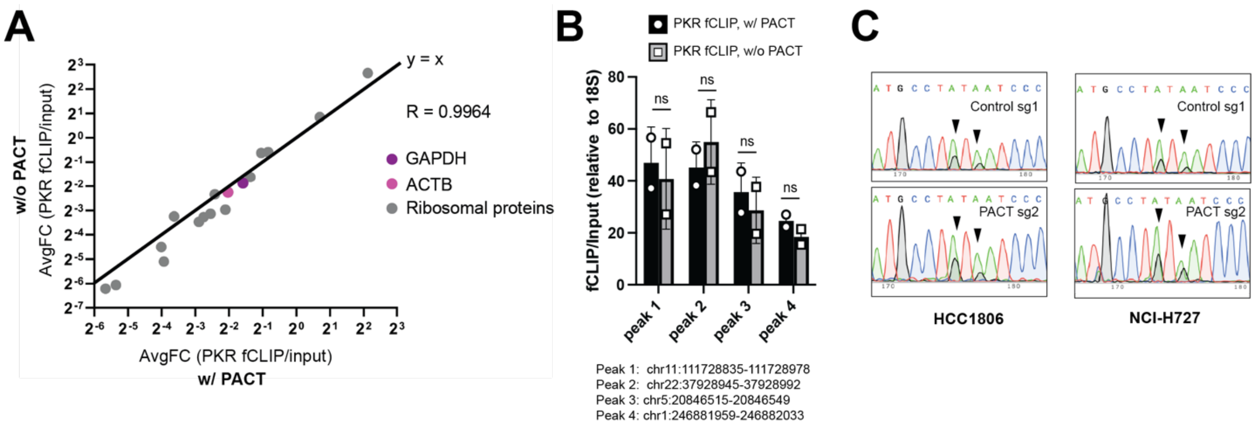

Supplementary Figure 4. PACT does not sequester endogenous dsRNA ligands away from PKR.

(A) Validation of the normalization strategies for fCLIP-seq in Figure 2. We examined 17 distinct, unrelated housekeeping genes—including ACTB, GAPDH, and various ribosomal protein genes— from HA-PKR fCLIP with and without PACT. While these genes do not harbor fCLIP peaks that meet the filtering criteria in Figure 2C, they still have read counts from HA-PKR fCLIP-seq, allowing us to assess the normalization strategies. The values were plotted using the same method as in Figure 2F. The results showed nearly identical PKR fCLIP-to-input fold change of house-keeping genes in the presence and absence of PACT (with the correlation coefficient R of 0.9964), suggesting equivalent background levels.

(B) Re-examination of 4 peaks selected from (Figure 2F) by fCLIP RT-qPCR. Experiments were performed as in Figure 2F. Values were obtained by first normalizing indicated RNA levels to the internal control 18S rRNA, and then calculating the ratio of fCLIP to input for the normalized RNA levels. The results represent mean ± SD from 2 independent experiments. *P*-values were calculated by two-tailed t-test. (ns, not significant; P>0.05).

(C) Representative sequencing chromatograms showing A-to-I editing sites in PHAX mRNA 3’UTR Alu sequence in control vs. PACT-KO in HCC1806 and NCI-H727 cells.

Source data and exact *P*-values are provided as a Source Data file.

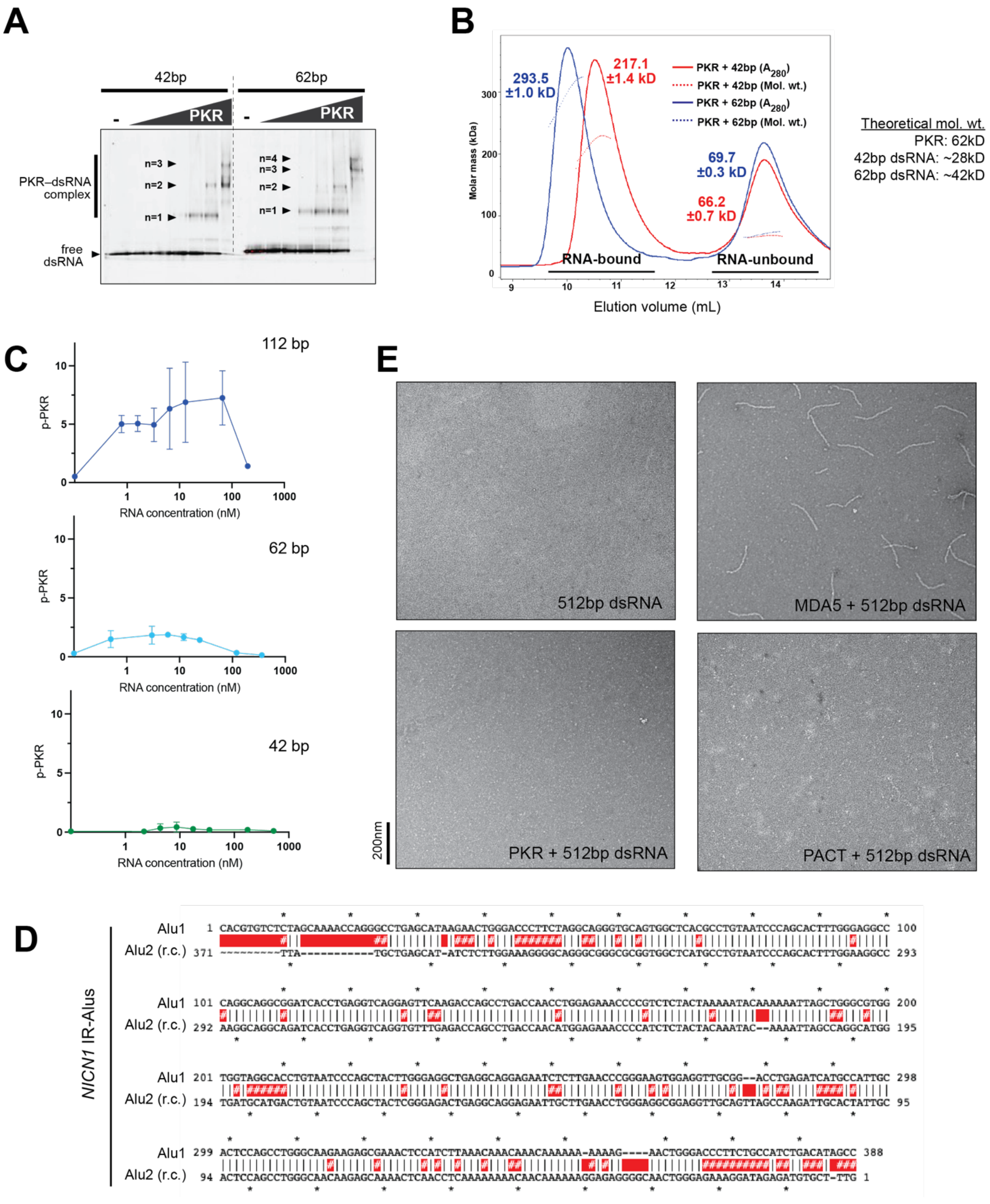

Supplementary Figure 5. PKR can measure dsRNA length beyond 30 bp without forming filaments.

(D) Native gel-shift assay showing binding of increasing concentrations (0, 78.1, 156.25, 312.5 625, 1250, 2500 nM) of PKR to 42 and 62 bp dsRNA (25 ng/μl). Gels were stained with Sybr

gold. The number of complex bands suggests that 42 and 62 bp dsRNA can be occupied by up to 3 and 4 PKR molecules, respectively.

(E) SEC-MALS analysis with PKR bound to 42 bp (red) and 62 bp (blue) dsRNA. Catalytic dead K296R mutant of PKR was used. Estimated molecular weights of the complexes are consistent with the notion that 42 bp and 62 bp dsRNAs are bound by 3 and 4 PKR molecules, respectively.

(F) *In vitro* PKR kinase assay results in Figure 3D, but shown as a function of molar concentrations of RNA, rather than mass concentration.

(G) Complementarity of *NICN1* IR-Alus, represented by alignment between the first Alu in the pair (Alu1) and reverse complement (r.c.) of the second Alu (Alu2). Vertical lines indicate complementarity, while red highlights indicate mismatches (#) or bulges (no symbol).

(H) Negative-stain EM of 512 bp dsRNA with and without 300 nM MDA5ΔCARDs, PKR, PACT. While MDA5 forms filaments along the length of dsRNA, PKR and PACT do not form similar assemblies. The results were reproduced in 3 independent replicates.

Source data are provided as a Source Data file.

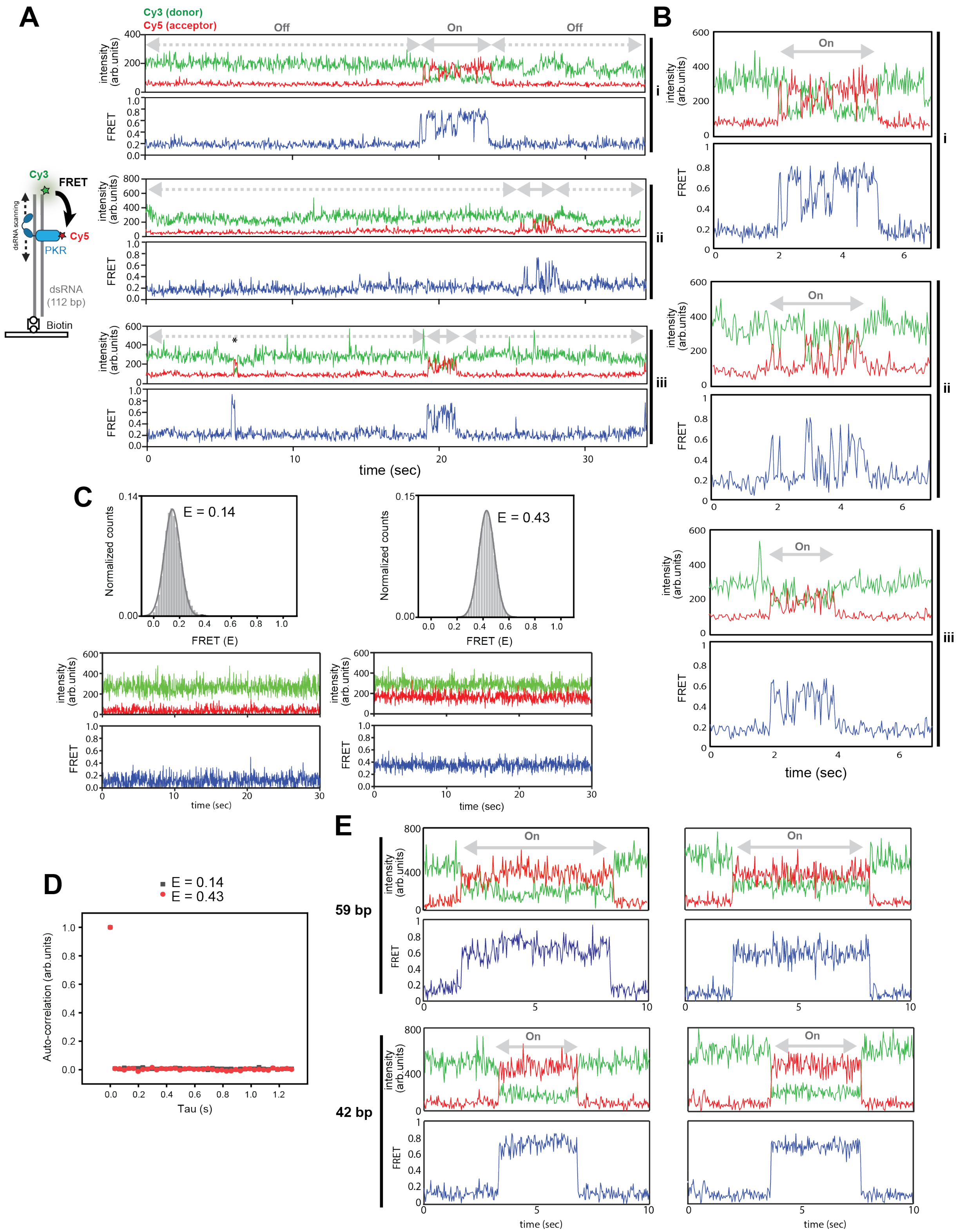

Supplementary Figure 6. PKR scans along dsRNA in a length-dependent manner.

(A) Schematic of single molecule FRET experiments to monitor scanning motions of PKR on dsRNA. Cy5-labeled PKR (acceptor, 50 nM) was added to a chamber with Cy3-labeled 112

bp dsRNA (donor) immobilized on the surface. Cy3 and Cy5 fluorescence signals were measured upon Cy3 excitation using sm-TIRF microscopy. 3 representative time course traces of Cy3/Cy5 intensities and FRET (right panel). PKR bound to dsRNA infrequently, typically exhibiting one or two binding events (FRET>~0.6 and anticorrelated Cy5 and Cy3 signal changes) over ~30 seconds. While some binding events were isolated, lasting < 2s (*), many occurred in clusters ("On"), during which rapid FRET fluctuations were observed, similar to those previously reported for TRBP and PACT[53,54]. The isolated events were classified as abortive and were excluded from the autocorrelation analysis. Similar classification has been adopted in previous studies[54,75]. PKR 296R was used to ensure analysis of PKR movement before its activation. Created in BioRender. torres, c. (2025) https://BioRender.com/g96x368.

(B) Zoomed in trace for "On" state in (A).

(C) Histograms and representative time traces of Cy3-labeled 112 bps dsRNA in the absence of any protein. The gaussian-fitted FRET value of the dsRNA and dsDNA showed E=0.14 and 0.43 respectively.

(D) Autocorrelation analysis of FRET signals for nucleic acids with constant E = 0.14 and E = 0.43 from (C). For stable structures without dynamic diffusion, the autocorrelation curve remains invariant, irrespective of E values. This is similar to that of the PKR-unbound state (off) but in contrast to a single exponential decay curve in PKR-bound state (on) in Fig. 3G.

(E) Representative time course traces of Cy3/Cy5 intensities and FRET with Cy5-labeled PKR (acceptor, 50 nM) and Cy3-labeled dsRNA (donor): 59 bp (top) and 42 bp (bottom).

Source data are provided as a Source Data file.

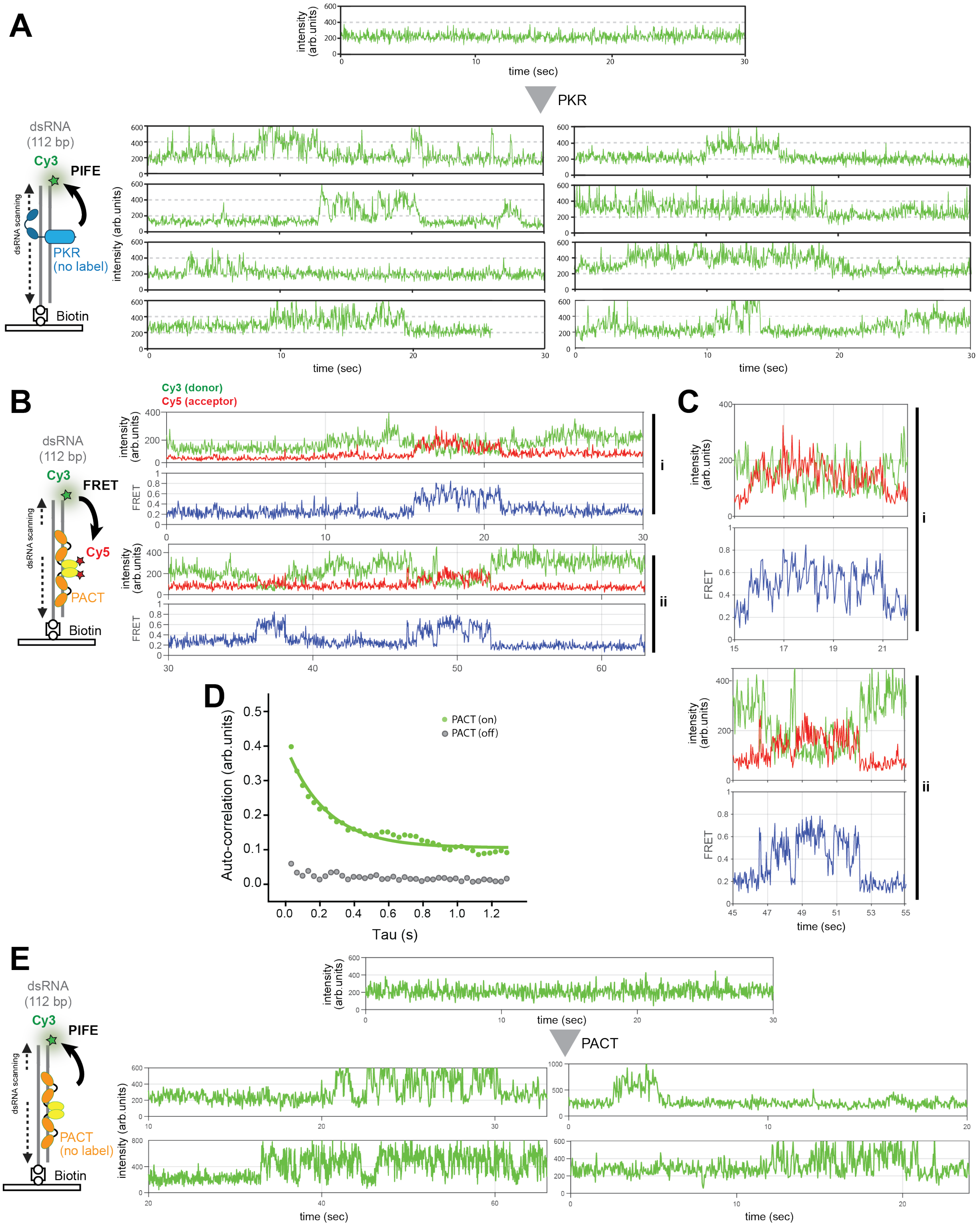

Supplementary Figure 7. Both PKR and PACT can scan along dsRNA.

(A) Schematic for PIFE visualization of PKR's diffusion on Cy3-labeled 112 bp dsRNA. Representative time traces before (top) and after (bottom) adding 10 nM unlabeled PKR to

Cy3-labeled 112 dsRNA. Repetitive Cy3 intensity fluctuations were observed upon addition of PKR. The intensity of Cy3 can increase only by proximal protein contacts. Thus, the rapid Cy3 intensity changes are consistent with the PKR's repetitive sliding along dsRNA, consistent with the FRET analysis in Figure 3. Created in BioRender. torres, c. (2025) https://BioRender.com/g96x368.

(B) Schematic of single molecule FRET experiments to monitor scanning motions of PACT on dsRNA. Cy5-labeled PACT (acceptor, 30 nM) was added to a chamber containing Cy3-labeled 112 bp dsRNA (donor) immobilized on the surface. Cy3 and Cy5 fluorescence were monitored upon Cy3 excitation using sm-TIRF microscopy. 2 representative time course traces of Cy3/Cy5 intensities and FRET (right panel). Created in BioRender. torres, c. (2025) https://BioRender.com/g96x368.

(C) Zoomed in trace for "On" state in (B).

(D) Autocorrelation analysis of FRET signal for PACT "On" (green) and "Off" (gray) states. Scanning times were calculated by single exponential curve fitting. 108 diffusion events were analyzed for each condition. See Methods for the autocorrelation function and exponential curve fitting.

(E) Schematic for PIFE visualization of PACT's diffusion on Cy3-labeled 112 bp dsRNA. Representative time traces before (top) and after (bottom) adding 10 nM unlabeled PACT to Cy3-labeled 112 dsRNA. Repetitive Cy3 intensity fluctuations were observed only upon adding PACT. The intensity of Cy3 can increase only by proximal protein contact, so the rapid intensity changes are consistent with the PACT's repetitive sliding along dsRNA, consistent with the FRET analysis in (B-D). Created in BioRender. torres, c. (2025) https://BioRender.com/g96x368.

Source data are provided as a Source Data file.

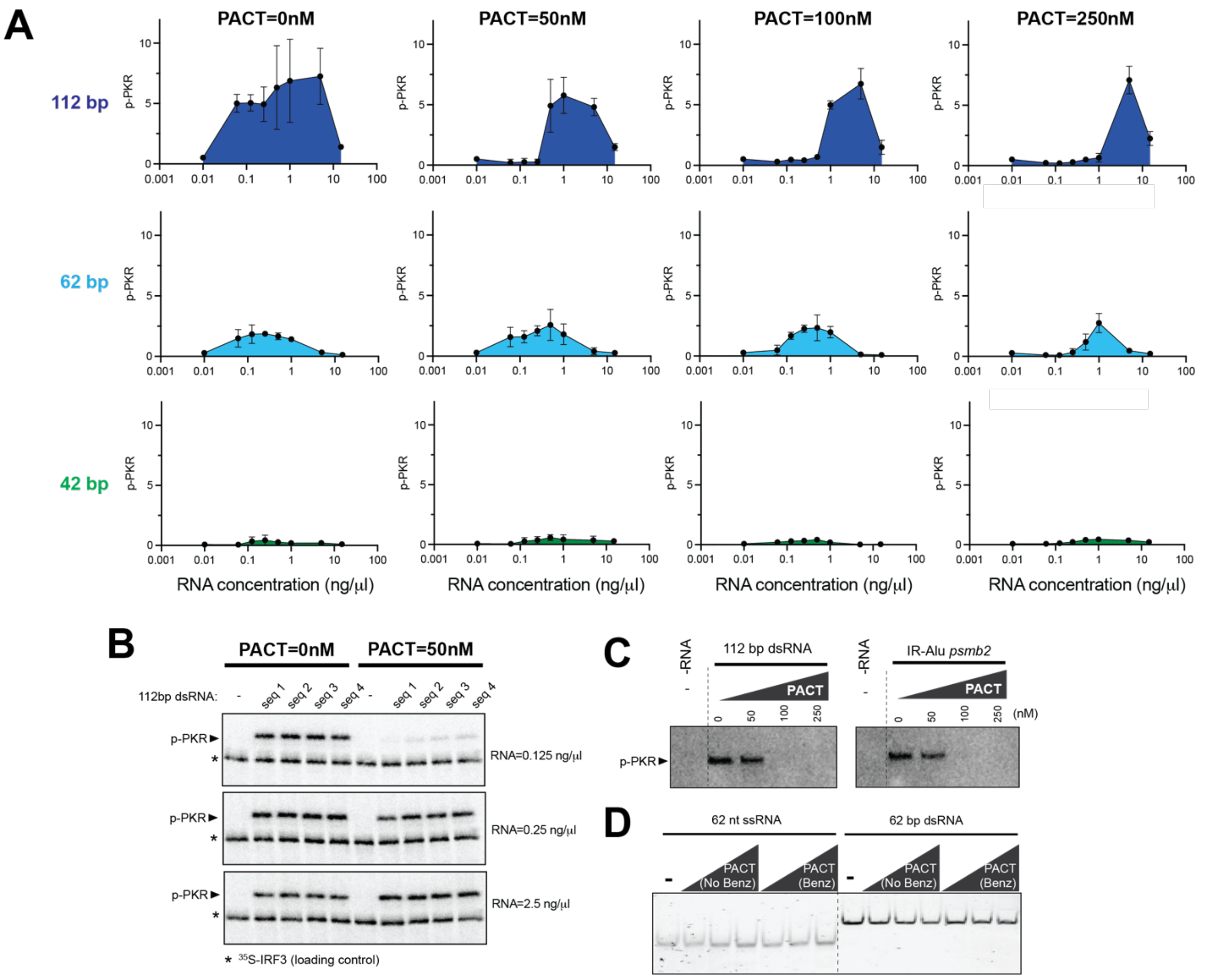

Supplementary Figure 8. PACT's ability to inhibit PKR is also dependent on dsRNA length.

(A) *In vitro* PKR kinase assay results from Figure 4A. Values represent means (±SD) of 3 biological repeats.

(B) *In vitro* PKR kinase assay using 112 bp dsRNA with 4 different sequences, seq 1 (GC%=52.7), seq 2 (GC%=50.1), seq 3 (GC%=42.9) and seq 4 (GC%=58.0) at 0.125. 0.25 and 2.5 ng/μl in the presence of 0, 50 nM PACT. See Supplementary table 3 for RNA sequences. Results without PACT was reproduced from Figure 3A.

(C) *In vitro* PKR kinase assay using 112 bp dsRNA or *PSMB2* IR-Alus (0.25 ng/μl) in the presence of 0, 50, 100, 250 nM PACT.

(D) Benzonase contamination test by incubating 62 nt ssRNA or 62 bp dsRNA with increasing concentration (50, 100, 200nM) of PACT that was either purified without benzonase treatment (No Benz) or with benzonase treatment (Benz). The incubation was done for 2 h at 37°C in the presence of 1.5 mM $MgCl_2$.

Source data are provided as a Source Data file.

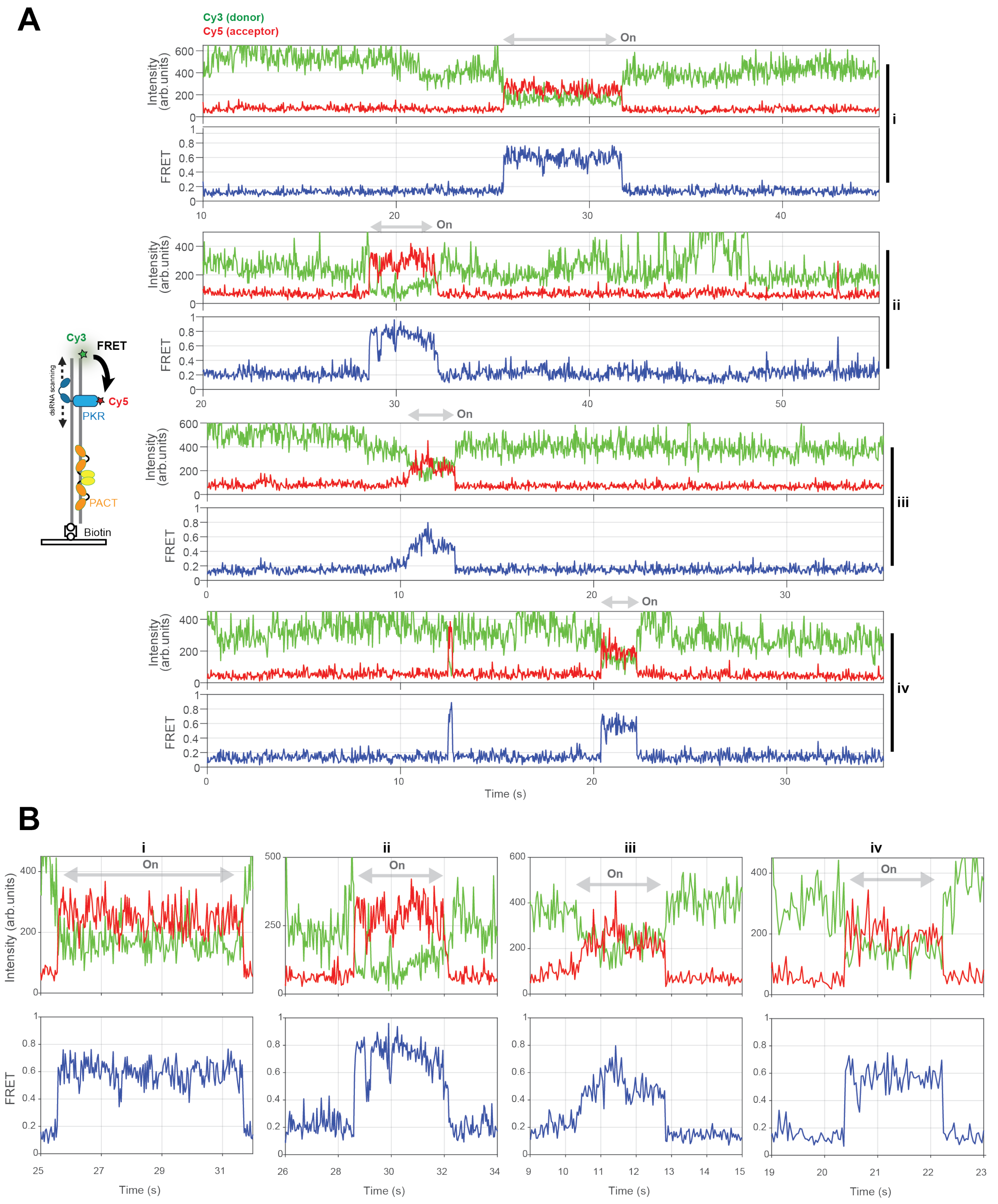

Supplementary Figure 9. PACT disrupts PKR scanning activity on dsRNA.

(A) Schematic of single molecule FRET experiments to monitor diffusion motions of PKR on dsRNA in the presence of PACT. Cy5-labeled PKR (acceptor, 50 nM) was mixed with unlabeled PACT (50 nM) and was added to a chamber containing Cy3-labeled dsRNA (donor)

immobilized on the surface. Cy3 and Cy5 fluorescence and Cy3-Cy5 FRET were monitored upon Cy3 excitation using sm-TIRF. 4 representative time course traces of Cy3/Cy5 intensities and FRET (right panel). PKR 296R was used to ensure analysis of PKR movement before its activation. Created in BioRender. torres, c. (2025) https://BioRender.com/g96x368.

(B) Zoomed in trace for “On” state in (A).

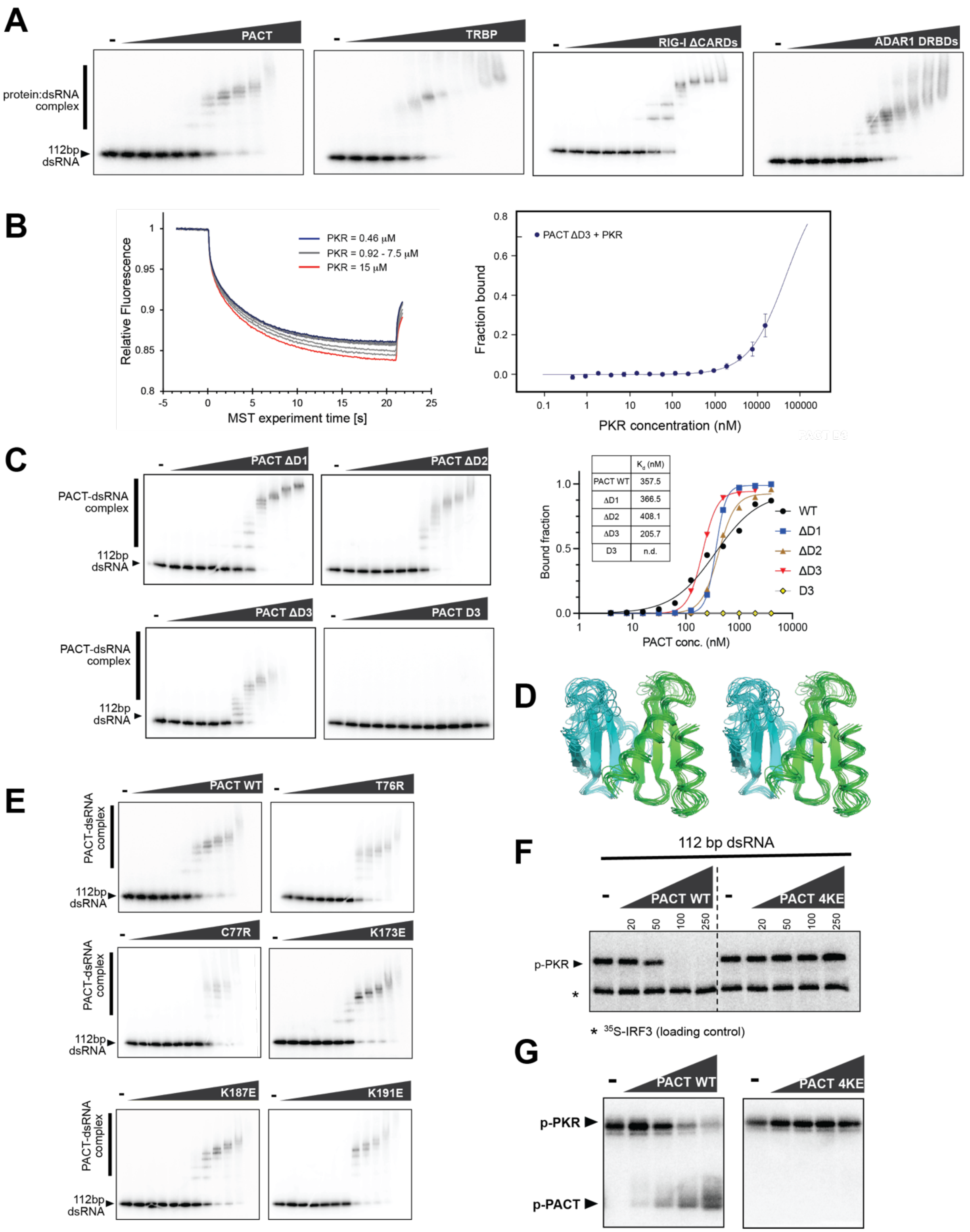

Supplementary Figure 10. PACT forms a direct interaction with PKR to inhibit its kinase activity.

(A) Native gel-shift assay monitoring 112 bp dsRNA binding to increasing concentrations (0, 3.9, 7.8, 15.6, 31.3, 62.5, 125, 250, 500, 1000, 2000, 4000 nM) of PACT, TRBP, RIG-I ΔCARDs, ADAR1 DRBDs. Binding curve analysis is shown in Figure 5B.

(B) Microscale thermophoresis analysis showing binding of GST-tagged PKR (K296R) to FAM-labelled PACT ΔD3. MST trace (left) and dose-response curve (right) with PACT ΔD3 titrated against PKR (0.000458 to 15 μM), from 3 independent experiments.

(C) Native gel-shift assay monitoring 112 bp dsRNA binding to increasing concentrations (0, 3.9, 7.8, 15.6, 31.3, 62.5, 125, 250, 500, 1000, 2000, 4000 nM) of PACT wild type, ΔD1, ΔD2, ΔD3, D3. Right: RNA binding curves.

(D) Stereo view of 15 superimposed lowest energy structures of PACT-D3 dimer.

(E) Native gel-shift assay monitoring 112 bp dsRNA binding to increasing concentrations (0, 3.9, 7.8, 15.6, 31.3, 62.5, 125, 250, 500, 1000, 2000, 4000 nM) of PACT wild type, T76R, C77R, K173E, K187E, K191E. Binding curve analysis is shown in Figure 6B.

(F) *In vitro* PKR kinase assay using 112 bp dsRNA (0.25 ng/μl) in the presence of 0, 20, 50, 100, 250 nM PACT WT and 4KE.

(G) RNA-independent PKR kinase assay with 1.5 μM PKR in the presence of 0, 1.25, 2.5, 5, 10 μM PACT WT and 4KE. Based on our results in Figure 6, where mutations in β3 of DRBD2 impair PACT functions while equivalent mutations in β3 of DRBD1 do not, we speculate that K84 and K85 of DRBD1, together with β3 of DRBD2, are involved in PKR binding. Consistent with this hypothesis, 4KE does not undergo phosphorylation by PKR, unlike WT PACT, suggesting an impaired PKR-PACT interaction.

Source data are provided as a Source Data file.

A

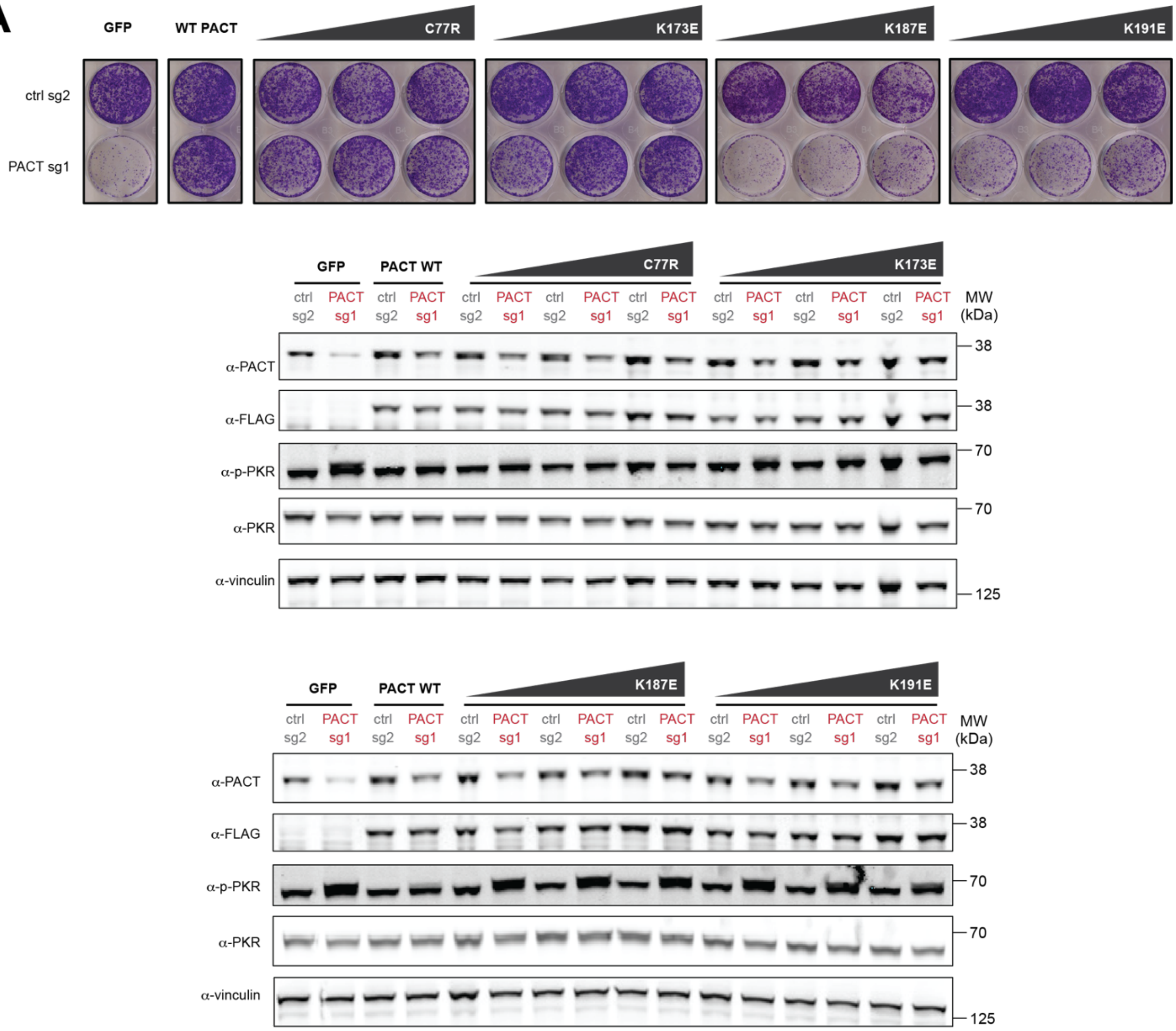
GFP
WT PACT
C77R
K173E
K187E
K191E
ctrl sg2
PACT sg1
GFP
PACT WT
C77R
K173E
ctrl sg2
PACT sg1
MW (kDa)
α-PACT
α-FLAG
α-p-PKR
α-PKR
α-vinculin
38
38
70
70
125
GFP
PACT WT
K187E
K191E
ctrl sg2
PACT sg1
MW (kDa)
α-PACT
α-FLAG
α-p-PKR
α-PKR
α-vinculin
38
38
70
70
125

B

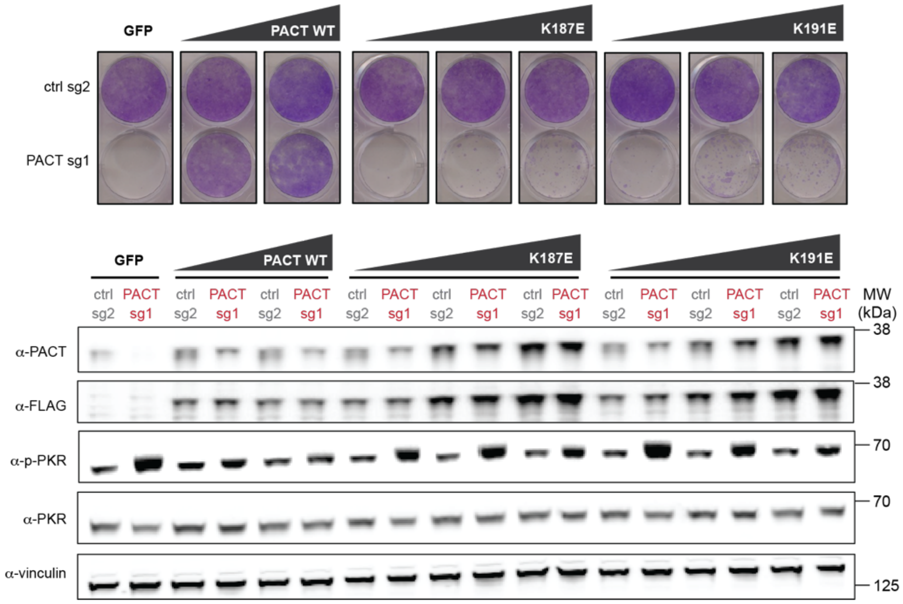
GFP
PACT WT
K187E
K191E
ctrl sg2
PACT sg1
GFP
PACT WT
K187E
K191E
ctrl sg2
PACT sg1
MW (kDa)
α-PACT
α-FLAG
α-p-PKR
α-PKR
α-vinculin
38
38
70
70
125

Supplementary Figure 11. PACT DRBD2 interface for PKR interaction is critical for PKR suppression.

(A) PACT complementation assay using crystal violet staining and Western blot analyses. NCI-H727 cells, either control gene-KO and PACT-KO, were complemented with increasing amounts of lentiviruses expressing GFP, Flag-tagged PACT wildtype, C77R, K173E, K187E or K191E.
(B) PACT complementation assay using crystal violet staining and Western blot analyses. HCC1806 cells, either control gene-KO and PACT-KO, were complemented with increasing amounts of lentiviruses expressing GFP, Flag-tagged PACT wildtype, K187E or K191E.

Source data are provided as a Source Data file.

Supplementary Table 1. NMR and refinement statistics.

**NMR distance and dihedral constraints** [a]	A (30-99)	B (30-99)
Distance constraints from NOE	202	198
Short-range intramolecular ($\|i-j\| \leq 4$)	139	145
Long-range intramolecular ($\|i-j\| \geq 5$)	38	28
Intermolecular	25	
Total dihedral angle restraints [b]	110	112
ϕ (TALOS)	55	56
ψ (TALOS)	55	56
Hydrogen bond restraints		
Intramolecular	18	18
Intermolecular	6	
**Structure statistics** [c]		
Violations (mean ± s.d.)		
Distance constraints (Å)	0.062 ± 0.004	
Dihedral angle constraints (°)	0.564 ± 0.028	
Deviations from idealized geometry		
Bond lengths (Å)	0.005 ± 0.000	
Bond angles (°)	0.626 ± 0.008	
Impropers (°)	0.466 ± 0.018	
Average pairwise r.m.s. deviation (Å) [d]		
Heavy	1.599	
Backbone	0.985	

[a] The numbers of constraints are summed over two subunits.

[b] Backbone ϕ and ψ restraints and their respective uncertainties were obtained from the "GOOD" dihedrals generated by the TALOS+ program based on the backbone chemical shift values.

[c] Statistics are calculated and averaged over an ensemble of the 15 lowest energy structures out of 75 calculated structures.

[d] The precision of the atomic coordinates is defined as the average r.m.s. difference between the 15 final structures and their mean coordinates except the flexible loop region (residue 52 to 59).

Supplementary Table 2. sgRNA sequences utilized for CRISPR-Cas9-mediated gene deletion. The targeted gene (column A), name of the sgRNA used in this study (column B), and sgRNA sequence (column C) are provided.

**Gene name**	**Targeting sgRNA name**	**sgRNA sequence**
AAVS	Control sg1	GAGCCACATTAACCGGCCCT
Chr2.2	Control sg2	GGTGTGCGTATGAAGCAGTG
PACT (PRKRA)	PACT sg1	CCAGGGAAAACACCGATTC
PACT (PRKRA)	PACT sg2	TCACCAACGGTTACTCTGA
PKR (EIF2AK2)	PKR sg1	GCAAGACTATGGAAAGGAAG
PKR (EIF2AK2)	PKR sg2	AAAGGCAATACGTACCACTG
MAVS	MAVS sg1	CCTCTCCTGGAACTTCCGGT
MAVS	MAVS sg2	GGTATTGAAGAGATGCCAGA
RNase L	RNase L sg1	ATCTTGTTATGACAGCGAGG
RNase L	RNase L sg2	GCGTGTTTGGATGTGCACAG

Supplementary Table 3. Sequences of dsRNAs used in the study.

dsRNA	Forward strand sequence	Reverse strand sequence
42 bp<br>dsRNA	GGGAGAAUGUCGAAUGGGUAUUCCACAGACGAGAAUUCUCCC	GGGAGAAUUCUCGUCUGUGGAAUACCCAUUCGACAUUCUCCC
62 bp<br>dsRNA	GGGAGAAUGUCGAAUGGGUAUUCCACAGACGAGAAUUUCCGCU<br>AUCUCAUCUCGUGUCUCCC	GGGAGACACGAGAUGAGAUAGCGGAAAUUCUCGUCUGUGGAAU<br>ACCCAUUCGACAUUCUCCC
112 bp<br>dsRNA<br>(seq 1)	GGGAGAAUGUCGAAUGGGUAUUCCACAGACGAGAAUUUCCGCU<br>AUCUCAUCUCGUGCUUCAGGGCCAGGGUGAAAAUGUACAUCCAG<br>GUGGAGCCUGUGCUGGACUUCUCCC	GGGAGAAGUCCAGCACAGGCUCCACCUGGAUGUACAUUUUCACC<br>CUGGCCCUGAAGCACGAGAUGAGAUAGCGGAAAUUCUCGUCUG<br>UGGAAUACCCAUUCGACAUUCUCCC
112 bp<br>dsRNA<br>(seq 2)	GGGAGAAUGAACACGAUUAACAUCGCUAAGAACGACUUCUCUGA<br>CAUCGAACUGGCUGCUAUCCCGUUCAACACUCUGGCUGACCAUU<br>ACGGUGAGCGUUUAGCUCUCUCCC	GGGAGAGAGCUAAACGCUCACCGUAAUGGUCAGCCAGAGUGUU<br>GAACGGGAUAGCAGCCAGUUCGAUGUCAGAGAAGUCGUUCUUA<br>GCGAUGUUAAUCGUGUUCAUUCUCCC
112 bp<br>dsRNA<br>(seq 3)	GGGAGAAAAAACGUUGAGGAACAACUCAACAAGCGCGUAGGGCA<br>CGUCUACAAGAAAGCAUUUAUGCAAGUUGUCGAGGCUGACAUG<br>CUCUCUAAUAUUAUAAUAAUCUCCC	GGGAGAUUAUUAUAAUAUUAGAGAGCAUGUCAGCCUCGACAAC<br>UUGCAUAAAUGCUUUCUUGUAGACGUGCCCUACGCGCUUGUUG<br>AGUUGUUCCUCAACGUUUUUUCUCCC
112 bp<br>dsRNA<br>(seq 4)	GGGAGAGCCGGCCGCCGUAGCGUACAUCACCAUUAAGACCACUC<br>UGGCUUGCCUAACCAGUGCUGACAAUACAACCGUUCAGGCUGUA<br>GCAAGCGCAAUCGGUCGGUCUCCC	GGGAGACCGACCGAUUGCGCUUGCUACAGCCUGAACGGUUGUA<br>UUGUCAGCACUGGUUAGGCAAGCCAGAGUGGUCUUAAUGGUGA<br>UGUACGCUACGGCGGCCGGCUCUCCC
512 bp<br>dsRNA	GGGAGAAUGUCGAAUGGGUAUUCCACAGACGAGAAUUUCCGCU<br>AUCUCAUCUCGUGCUUCAGGGCCAGGGUGAAAAUGUACAUCCAG<br>GUGGAGCCUGUGCUGGACUACCUGACCUUUCUGCCUGCAGAGG<br>UGAAGGAGCAGAUUCAGAGGACAGUCGCCACCUCCGGGAACAUG<br>CAGGCAGUUGAACUGCUGCUGAGCACCUUGGAGAAGGGAGUCU<br>GGCACCUUGGUUGGACUCGGGAAUUCGUGGAGGCCCUCCGGAG<br>AACCGGCAGCCCUCUGGCCGCCCGCUACAUGAACCCUGAGCUCAC<br>GGACUUGCCCUCUCCAUCGUUUGAGAACGCUCAUGAUGAAUAUC<br>UCCAACUGCUGAACCUCCUUCAGCCCACUCUGGUGGACAAGCUU<br>CUAGUUAGAGACGUCUUGGAUAAGUGCAUGGAGGAGGAACUGU<br>UGACAAUUGAAGACAGAAACCGGAUUGCUGCUGCAGAAAACAAU<br>GGAAAUGAAUCAGGUGUAAGAGAGCUUCUCCC	GGGAGAAGCUCUCUUACACCUGAUUCAUUUCCAUUGUUUUCUG<br>CAGCAGCAAUCCGGUUUCUGUCUUCAAUUGUCAACAGUUCCUCC<br>UCCAUGCACUUAUCCAAGACGUCUCUAACUAGAAGCUUGUCCAC<br>CAGAGUGGGCUGAAGGAGGUUCAGCAGUUGGAGAUAUUCAUCA<br>UGAGCGUUCUCAAACGAUGGAGAGGGCAAGUCCGUGAGCUCAG<br>GGUUCAUGUAGCGGGCGGCCAGAGGGCUGCCGGUUCUCCGGAG<br>GGCCUCCACGAAUUCCCGAGUCCAACCAAGGUGCCAGACUCCCUU<br>CUCCAAGGUGCUCAGCAGCAGUUCAACUGCCUGCAUGUUCCCGG<br>AGGUGGCGACUGUCCUCUGAAUCUGCUCCUUCACCUCUGCAGGC<br>AGAAAGGUCAGGUAGUCCAGCACAGGCUCCACCUGGAUGUACAU<br>UUUCACCCUGGCCCUGAAGCACGAGAUGAGAUAGCGGAAAUUCU<br>CGUCUGUGGAAUACCCAUUCGACAUUCUCCC
IR-Alu<br>*NICN1*	CACGUGUCUCUAGCAAAACCAGGGCCUGAGCAUAAGAACUGGGA<br>CCCUUCUAGGCAGGGUGCAGUGGCUCACGCCUGUAAUCCCAGCA<br>CUUUGGGAGGCCCAGGCAGGCGGAUCACCUGAGGUCAGGAGUU<br>CAAGACCAGCCUGACCAACCUGGAGAAACCCCGUCUCUACUAAAA<br>AUACAAAAAAUUAGCUGGGCGUGGUGGUAGGCACCUGUAAUCC<br>CAGCUACUUGGGAGGCUGAGGCAGGAGAAUCUCUUGAACCCGG<br>GAAGUGGAGGUUGCGGACCUGAGAUCAUGCCAUUGCACUCCAGC<br>CUGGGCAAGAAGAGCGAAACUCCAUCUUAAACAAACAAACAAAA<br>AAAAAAGAACUGGGACCCUUCUGCCAUCUGACAUAGCC	CAAAGCACAUCUCUAUCCUUUCUCCCAGUUGCCCCUCUCCUUUU<br>UUGUUGUUUUUUUUGAGGUUGAGUUUUGCUCUUGUUGCCCAG<br>GCUGGAGUGCAAUAGUGCAAUCUUGGCUAACUGCAACCUCCGCC<br>UCCCAGGUUCAAGCAAUUCUCCUGCCUCAGUCUCCCGAGUAGCU<br>GGGAUUACAGUCAUGCAUCACCAUGCCUGGCUAAUUUUGUAUU<br>UGUAGUAGAGAUGGGGUUUCUCCAUGUUGGUCAGGCUGGUCU<br>CAAACACCUGACCUCAGGUGAUCUGCCUGCCUUGGCCUUCCAAA<br>GUGCUGGGAUUACAGGCAUGAGCCACCGCGCCCGCCCUGCCCCU<br>UUCCAAGAGAUAUGCUCAGCAUAA
IR-Alu<br>*PSMB2*	GAAGCAGGAAGCAGAUGACUGGAUUAUAGGGAUGGAAGAAACA<br>UUUUUCCUUCUUUUUUUGAGACGGAGUCUCACUCUGUUGCCAG<br>GCUGGAGUACAGUGGCGCGAUCUAGGCUCACUGCAACCUCUGCC<br>UCCCGGGUUCGACCGAUUCUCCUGUCUCAGCCUCCCCAGUAGCU<br>GGGACUAGGGGUGCGUGCCACCACGCCCAGCUAAUUUUUGUAU<br>UUUUAGUAGAGACGGGGUUUUACCAUGUUGGUCAGGAUGGUC<br>UCGCUCUCUUGACCUCGUGAUCCGCCCGCCUCGGCCUCCCAAAG<br>UGCUGGGAUUACAGGUAUGAGCCACCACACCCAGCUCUGGAAGA<br>AAUAUUUUUCACUACAUGUUUUUGUACUGUUUGCAUGUUACAU<br>UAA	CAAUCUCUAAUAUAGUGUUAAGUGAAAUAAGCAAUAUGCAGAA<br>CUUAAAAAUACUUUUUAGCCGGGCACGGUGGCUCACGCCUGUAA<br>UCCCAGCACUGGGAAGCCAAGGCAGGCAGACUGCUUGAGCCCAG<br>GAGUUCAAGACCAACCUGAAUGACAUGGUGAGACCCUGUCUCUA<br>UGAAAAACACAAAAACUAGCUGGGCAUUGGUGGUGUGCGCCUG<br>UAAUCCUAGCUACUCAGGAGGCUGAGAUGGGAGGAUCACUUGG<br>GCCCGGGAGGCGGAGGUUGCAGUGAGCCAAGAUUACGCCACAGC<br>ACUCCAGCCUGGGCUAGAGUGAGACCCUGUCUCUAAAUAAAUAA<br>AUAAAUCUAAAAAAUUUAAAGGGGGCAUGUGUCUGUAUGUAUG<br>UAUA
Alu<br>dsRNA	GGCUAUGUCAGAUGGCAGAAGGGUCCCAGUUCUUUUUUUUUU<br>GUUUGUUUGUUUAAGAUGGAGUUUCGCUCUUCUUGCCCAGGC<br>UGGAGUGCAAUGGCAUGAUCUCAGGUCCGCAACCUCCACUUCCC<br>GGGUUCAAGAGAUUCUCCUGCCUCAGCCUCCCAAGUAGCUGGGA<br>UUACAGGUGCCUACCACCACGCCCAGCUAAUUUUUUGUAUUUU<br>UAGUAGAGACGGGGUUUCUCCAGGUUGGUCAGGCUGGUCUUGA<br>ACUCCUGACCUCAGGUGAUCCGCCUGCCUGGGCCUCCCAAAGUG<br>CUGGGAUUACAGGCGUGAGCCACUGCACCCUGCCUAGAAGGGUC<br>CCAGUUCUUAUGCUCAGGCCCUGGUUUUGCUAGAGACACGUG	CACGUGUCUCUAGCAAAACCAGGGCCUGAGCAUAAGAACUGGGA<br>CCCUUCUAGGCAGGGUGCAGUGGCUCACGCCUGUAAUCCCAGCA<br>CUUUGGGAGGCCCAGGCAGGCGGAUCACCUGAGGUCAGGAGUU<br>CAAGACCAGCCUGACCAACCUGGAGAAACCCCGUCUCUACUAAAA<br>AUACAAAAAAUUAGCUGGGCGUGGUGGUAGGCACCUGUAAUCC<br>CAGCUACUUGGGAGGCUGAGGCAGGAGAAUCUCUUGAACCCGG<br>GAAGUGGAGGUUGCGGACCUGAGAUCAUGCCAUUGCACUCCAGC<br>CUGGGCAAGAAGAGCGAAACUCCAUCUUAAACAAACAAACAAAA<br>AAAAAAGAACUGGGACCCUUCUGCCAUCUGACAUAGCC

Supplementary References

(75) Fareh, M. *et al.* TRBP ensures efficient Dicer processing of precursor microRNA in RNA-crowded environments. *Nat Commun* **7**, 13694 (2016). https://doi.org/10.1038/ncomms13694
