# Supplementary material for: PACT prevents aberrant activation of PKR by endogenous dsRNA without sequestration
Source: Nat Commun. 2025 Apr 8;16:3325. doi: 10.1038/s41467-025-58433-x (PMC11978871; doi:10.1038/s41467-025-58433-x)
Supplement: Supplementary file 2 — Description of Additional Supplementary Files [file 41467_2025_58433_MOESM2_ESM.pdf]

File name: Supplementary Data 1.

Description: List of peaks identified from PKR fCLIP-seq. *P*-values were calculated in Deseq2 (two-sided) and adjusted by Benjamini-Hochberg method.

File name: Supplementary Data 2.

Description: List of enriched peaks in PKR fCLIP-seq. Enriched peaks were defined using criteria: (Log_2FC (HA-fCLIP/input) ≥1 in HA-PKR expressing cells & Log_2FC (HA-fCLIP/input) ≤0 without HA-PKR. *P*-values were calculated in Deseq2 (two-sided) and adjusted by Benjamini-Hochberg method.

File name: Supplementary Data 3.

Description: List of peaks identified from PACT fCLIP-seq. *P*-values were calculated in Deseq2 (two-sided) and adjusted by Benjamini-Hochberg method.

File name: Supplementary Data 4.

Description: List of enriched peaks in PACT fCLIP-seq. Enriched peaks were defined using criteria: (Log_2FC (FLAG-fCLIP/input) ≥1 in FLAG-PACT expressing cells & Log_2FC (FLAG-fCLIP/input) ≤0 without FLAG-PACT. *P*-values were calculated in Deseq2 (two-sided) and adjusted by Benjamini-Hochberg method.

File name: Supplementary Data 5.

Description: List of enriched peaks in PKR fCLIP-seq from PACT expressing and PACT knockout backgrounds. The AuC for each peak for all samples is provided (see Methods for details).
