# Supplementary material for: PACT prevents aberrant activation of PKR by endogenous dsRNA without sequestration
Source: Nat Commun. 2025 Apr 8;16:3325. doi: 10.1038/s41467-025-58433-x (PMC11978871; doi:10.1038/s41467-025-58433-x)
Supplement: Supplementary file 8 — Reporting Summary [file 41467_2025_58433_MOESM8_ESM.pdf]

Corresponding author(s): Sun Hur
Last updated by author(s): Mar 18, 2025

Reporting Summary

Nature Portfolio wishes to improve the reproducibility of the work that we publish. This form provides structure for consistency and transparency in reporting. For further information on Nature Portfolio policies, see our Editorial Policies and the Editorial Policy Checklist.

Statistics

For all statistical analyses, confirm that the following items are present in the figure legend, table legend, main text, or Methods section.

n/a	Confirmed	
☐	☒	The exact sample size (*n*) for each experimental group/condition, given as a discrete number and unit of measurement
☐	☒	A statement on whether measurements were taken from distinct samples or whether the same sample was measured repeatedly
☐	☒	The statistical test(s) used AND whether they are one- or two-sided<br>*Only common tests should be described solely by name; describe more complex techniques in the Methods section.*
☐	☒	A description of all covariates tested
☒	☐	A description of any assumptions or corrections, such as tests of normality and adjustment for multiple comparisons
☐	☒	A full description of the statistical parameters including central tendency (e.g. means) or other basic estimates (e.g. regression coefficient) AND variation (e.g. standard deviation) or associated estimates of uncertainty (e.g. confidence intervals)
☐	☒	For null hypothesis testing, the test statistic (e.g. *F*, *t*, *r*) with confidence intervals, effect sizes, degrees of freedom and *P* value noted<br>*Give P values as exact values whenever suitable.*
☒	☐	For Bayesian analysis, information on the choice of priors and Markov chain Monte Carlo settings
☒	☐	For hierarchical and complex designs, identification of the appropriate level for tests and full reporting of outcomes
☒	☐	Estimates of effect sizes (e.g. Cohen's *d*, Pearson's *r*), indicating how they were calculated

Our web collection on statistics for biologists contains articles on many of the points above.

Software and code

Policy information about availability of computer code

Data collection	MO.Affinity Analysis v2.3 for MST data collection<br>NMRPipe for NMR data processing<br>XEASY and CcpNmr for NMR spectra analysis<br>XPLOR-NIH for NMR structure calculation<br>TALOS+ for calculation of protein backbone dihedral angles<br>smCamera for recording smFRET data

Data analysis	Prism10 for data plotting and statistical analyses<br>trimmomatic (v0.36) for trimming sequence data<br>SAMtools (v1.9) for sequence data alignment<br>bedtools genomecov (v2.30.0) for converting bam files to bedgraph files<br>macs2 (v2.1.1.20160309) bdgcmp for bedgraph subtraction<br>macs2 bdgpeakcall for peak-calling<br>bedtools (v2.30.0) for calculating AuC<br>Deseq2 for p-value and FC calculations for fCLIP-seq data<br>Code Availability:<br>Github link: https://github.com/DylannnWX/Hurlab_PKR_manuscript/tree/main

For manuscripts utilizing custom algorithms or software that are central to the research but not yet described in published literature, software must be made available to editors and reviewers. We strongly encourage code deposition in a community repository (e.g. GitHub). See the Nature Portfolio guidelines for submitting code & software for further information.

Data

Policy information about availability of data

All manuscripts must include a data availability statement. This statement should provide the following information, where applicable:

- Accession codes, unique identifiers, or web links for publicly available datasets
- A description of any restrictions on data availability
- For clinical datasets or third party data, please ensure that the statement adheres to our policy

Sequencing data is available at NCBI GEO dataset: GSE269684
NMR data is available at Biological Magnetic Resonance Bank: 36671
PDB coordinates are available at RCSB: 8ZU6

Research involving human participants, their data, or biological material

Policy information about studies with human participants or human data. See also policy information about sex, gender (identity/presentation), and sexual orientation and race, ethnicity and racism.

Reporting on sex and gender	*Use the terms sex (biological attribute) and gender (shaped by social and cultural circumstances) carefully in order to avoid confusing both terms. Indicate if findings apply to only one sex or gender; describe whether sex and gender were considered in study design; whether sex and/or gender was determined based on self-reporting or assigned and methods used.<br>Provide in the source data disaggregated sex and gender data, where this information has been collected, and if consent has been obtained for sharing of individual-level data; provide overall numbers in this Reporting Summary. Please state if this information has not been collected.<br>Report sex- and gender-based analyses where performed, justify reasons for lack of sex- and gender-based analysis.*
Reporting on race, ethnicity, or other socially relevant groupings	*Please specify the socially constructed or socially relevant categorization variable(s) used in your manuscript and explain why they were used. Please note that such variables should not be used as proxies for other socially constructed/relevant variables (for example, race or ethnicity should not be used as a proxy for socioeconomic status).<br>Provide clear definitions of the relevant terms used, how they were provided (by the participants/respondents, the researchers, or third parties), and the method(s) used to classify people into the different categories (e.g. self-report, census or administrative data, social media data, etc.)<br>Please provide details about how you controlled for confounding variables in your analyses.*
Population characteristics	*Describe the covariate-relevant population characteristics of the human research participants (e.g. age, genotypic information, past and current diagnosis and treatment categories). If you filled out the behavioural & social sciences study design questions and have nothing to add here, write "See above."*
Recruitment	*Describe how participants were recruited. Outline any potential self-selection bias or other biases that may be present and how these are likely to impact results.*
Ethics oversight	*Identify the organization(s) that approved the study protocol.*

Note that full information on the approval of the study protocol must also be provided in the manuscript.

Field-specific reporting

Please select the one below that is the best fit for your research. If you are not sure, read the appropriate sections before making your selection.

☒ Life sciences ☐ Behavioural & social sciences ☐ Ecological, evolutionary & environmental sciences

For a reference copy of the document with all sections, see nature.com/documents/nr-reporting-summary-flat.pdf

Life sciences study design

All studies must disclose on these points even when the disclosure is negative.

Sample size	Sample sizes varied depending on experiments and are noted in the figure legends and Methods.
Data exclusions	For fCLIP-seq analyses, please see Methods for details of excluding background peaks.
Replication	All experiments were performed at least 2-5 times. Only experimental data that were successfully replicated in all attempts are reported. See Figure legends for details.
Randomization	Experiments in this study were not subjected to randomization as the identity of the samples are predetermined during experiments; the experimental results would not be interpretable if these samples were randomized.
Blinding	For all the experiments conducted in this study, there were no samples that could be blinded as the identity of the samples are predetermined during experiments.

Reporting for specific materials, systems and methods

We require information from authors about some types of materials, experimental systems and methods used in many studies. Here, indicate whether each material, system or method listed is relevant to your study. If you are not sure if a list item applies to your research, read the appropriate section before selecting a response.

Materials & experimental systems

n/a	Involved in the study	
☐	☒	Antibodies
☐	☒	Eukaryotic cell lines
☒	☐	Palaeontology and archaeology
☒	☐	Animals and other organisms
☒	☐	Clinical data
☒	☐	Dual use research of concern
☒	☐	Plants

Methods

n/a	Involved in the study	
☒	☐	ChIP-seq
☒	☐	Flow cytometry
☒	☐	MRI-based neuroimaging

Antibodies

Antibodies used	Rabbit monoclonal anti-phospho-PKR Thr446 Abcam Cat # ab32036<br>Rabbit monoclonal anti-RNase L antibody Abcam Cat # ab191392<br>Rabbit monoclonal anti-phospho-eIF2 alpha Cell Signaling Technology Cat # 3398<br>Rabbit monoclonal anti-total eIF2 alpha Cell Signaling Technology Cat # 5324<br>Rabbit polyclonal anti-MAVS Cell Signaling Technology Cat # 3993<br>Rabbit polyclonal anti-total PKR Cell Signaling Technology Cat # 3072<br>Mouse monoclonal anti-β-actin Santa Cruz Biotechnology Cat # sc-47778<br>Mouse monoclonal anti-vinculin Thermo Fisher Scientific Cat # MA5-11690<br>Mouse monoclonal anti-PACT Santa Cruz Biotechnology Cat# sc-377103<br>Rabbit polyclonal anti-GST Cell Signaling Technology Cat # 2622<br>Rabbit monoclonal anti-HA tag Cell Signaling Technology Cat # 3724<br>Rabbit monoclonal anti-PKR Cell Signaling Technology Cat # 12297<br>Mouse monoclonal anti-Flag M2 Millipore Sigma Cat # F1804<br>Goat anti-mouse IRDye 680LT LI-COR Biosciences Cat # 926-68020<br>Goat anti-rabbit IRDye 800CW LI-COR Biosciences Cat # 926-32211
Validation	All primary antibodies were validated previously by the manufacturer. Citations of studies using these antibodies and user ratings are provided on the manufacturer's websites

Eukaryotic cell lines

Policy information about cell lines and Sex and Gender in Research

Cell line source(s)	ZR-75-1 ATCC CRL-1500<br>HCC1428 ATCC CRL-2327<br>NCI-H1437 ATCC CRL-5872<br>NCI-H2023 ATCC CRL-5912<br>NCI-H1650 ATCC CRL-5883

	HCC1806 ATCC CRL-2335<br>MDA-MB-157 ATCC HTB-24<br>NCI-H2286 ATCC CRL-5938<br>NCI-H727 ATCC CRL-5815<br>SW1271 ATCC CRL-2177
Authentication	none of the cell lines were authenticated
Mycoplasma contamination	All cell lines tested negative for Mycoplasma contamination
Commonly misidentified lines<br>(See ICLAC register)	No commonly misidentified cell lines were used in the study.

Plants

Seed stocks	*Report on the source of all seed stocks or other plant material used. If applicable, state the seed stock centre and catalogue number. If plant specimens were collected from the field, describe the collection location, date and sampling procedures.*
Novel plant genotypes	*Describe the methods by which all novel plant genotypes were produced. This includes those generated by transgenic approaches, gene editing, chemical/radiation-based mutagenesis and hybridization. For transgenic lines, describe the transformation method, the number of independent lines analyzed and the generation upon which experiments were performed. For gene-edited lines, describe the editor used, the endogenous sequence targeted for editing, the targeting guide RNA sequence (if applicable) and how the editor was applied.*
Authentication	*Describe any authentication procedures for each seed stock used or novel genotype generated. Describe any experiments used to assess the effect of a mutation and, where applicable, how potential secondary effects (e.g. second site T-DNA insertions, mosiacism, off-target gene editing) were examined.*
